# Preparation of a Wear-Resistant, Superhydrophobic SiO_2_/Polymethyl Methacrylate Composite Coating on Aluminum Surface Processed with Nanosecond Laser

**DOI:** 10.3390/ma16196485

**Published:** 2023-09-29

**Authors:** Jiyi Sun, Lin Zhu, Zhuang Liu

**Affiliations:** College of Light Industry, Harbin University of Commerce, Harbin 150028, China; yicheng0930@163.com

**Keywords:** nanosecond laser, superhydrophobic, self-cleaning, anti-corrosion, wear-resistant

## Abstract

Superhydrophobic coatings are limited by complex preparation processes and poor mechanical durability in practical applications. In this study, a mechanically robust superhydrophobic composite coating was applied to an aluminum surface that underwent processing with a nanosecond laser (referred to as a superhydrophobic aluminum surface). It exhibits a high water contact angle (WCA) of 158.81°, a low sliding angle (SA) of less than 5°, and excellent self-cleaning ability. The wear test shows its durability, and the corrosion test shows its excellent corrosion resistance. This study provides a framework for the preparation of robust superhydrophobic surfaces that may have potential applications in many fields.

## 1. Introduction

Superhydrophobic surfaces are those that exhibit a water-repellent property with a WCA greater than 150° and an SA hysteresis less than 10°. This non-wettability characteristic has a wide range of applications in numerous fields, such as condensation heat transfer, anti-icing, self-cleaning, drag reduction, and corrosion resistance, among others [1,2,3,4,5]. The most important features of superhydrophobic surfaces are the micro/nano multi-scale surface topography and the low surface energy of the surface material.

In recent years, superhydrophobic surfaces have been extensively studied. Li et al. [6] prepared hydrophobic stearate particles and suspended them in ethanol. The solution was then sprayed onto a stainless-steel surface, forming a highly corrosion-resistant superhydrophobic coating. Mostag-himi et al. [7] achieved superhydrophobicity by spraying rare earth oxides with hydrophobic properties onto a steel surface with a WCA of 164°. However, due to the high cost and scarcity of rare earth metals, this approach cannot be widely used in practical production. Wang et al. [8] deposited nickel nanoparticles onto an etched porous aluminum substrate by immersing it in a mixed solution containing lactic acid, NaH_2_PO_2_·H_2_O, CH_3_COONa, NaHCO_3_, and NiSO_4_·6H_2_O, followed by modification with 1H,1H,2H,2H-perfluorodecyltriethoxysilane to achieve a superhydrophobic nickel surface with a WCA of 164 + 2°. Qian et al. [9] proposed to use of the acid etching method to corrode the surfaces of metal aluminum, copper, and zinc to produce rough micro- and nanostructures and then to chemically modify the corroded metal surfaces with fluoroalkylsilane to achieve superhydrophobic metal surfaces. In recent years, various methods have been developed to prepare superhydrophobic surfaces with superior performance [10,11,12,13,14,15,16,17,18].

However, the features of superhydrophobic surfaces are highly susceptible to damage due to mechanical actions, which can lead to the loss of their superhydrophobic properties. In practical application environments, mechanical actions, such as collisions, touch, wind erosion, and rain erosion, are all unavoidable. The mechanical weakness greatly reduces the stability and reliability of superhydrophobic surfaces in practical applications. In recent years, there has been increasing attention given to how to enhance the mechanical properties of superhydrophobic surfaces [19]. The fragile micro- and nanostructures on superhydrophobic surfaces are easily destroyed under mechanical stress. The resulting Cassie–Baxter states where the grooves of the surface are not wetted by water to the Wenzel states where the grooves of the surface are wetted by water lead to the loss of the superhydrophobic function. Numerous studies have shown that the fundamental challenges of superhydrophobic surface and coating applications are due to the lack of mechanical stability in the micro–nano-scale system structure and the low surface energy stability [20]. To address these two issues, people have developed self-healing methods [21,22,23], composite coatings [24,25], and hierarchical rough structures to prepare superhydrophobic surfaces. Compared with other methods, a superhydrophobic surface prepared by these methods has higher stability and durability through increasing the strength of the surface coating bonding, releasing hydrophobic components after damage, and embedding nanoparticles in the micron structure.

There are numerous surface treatment methods available to enhance the adhesive performance of coatings, including sandpaper polishing, sandblasting, and chemical cleaning. Among these techniques, sandpaper polishing and sandblasting effectively improve the bonding quality by augmenting the roughness of the substrate surface [26]. However, manual sandpaper polishing exhibits a limited impact on surface roughness due to human variability and yields inconsistent results. With sandblasting, an additional layer of structure is introduced, making it important to ensure strong adhesion between the blasting material and the substrate. On the other hand, laser etching offers a simple and reliable approach to creating micro–nano-level structures on material surfaces for enhancing roughness through the adjustment of various process parameters. Additionally, it provides long-term stability and environmental friendliness [27,28].

This study aims to integrate the advantages of laser etching and coating methods to create a hierarchical micro/nanostructured composite surface, enhancing the mechanical properties of superhydrophobic surfaces. First, a micro-groove was formed on the aluminum surface using laser technology, and then the PMMA connection layer was impregnated on the aluminum surface. Finally, the nano-silica hydrophobic structure was filled via the impregnation method. The micron-scale structure based on laser etching and the PMMA connection layer effectively protected the internal nanostructure and significantly improved the mechanical properties of the surface. The challenge of creating superhydrophobic surfaces with excellent mechanical properties has been a problem for many researchers. By integrating the advantages of laser etching and coating methods, this study provides a reference for how to create durable, strong, and excellent superhydrophobic surfaces with improved mechanical properties.

## 2. Experiments and Methods

### 2.1. Materials and Pretreatment

The 10 mm × 10 mm × 1 mm 1060 aluminum (99.60%) was obtained from Shenzhen Hongtai Hardware Processing Co., Ltd. in Huizhou, China, with specific composition as shown in Table 1. Prior to use, it was polished with 800- and 2000-grit sandpaper and sequentially cleaned with anhydrous ethanol and deionized water for 15 min each, then naturally dried. The anhydrous ethanol was procured from Tianjin Zhiyuan Chemical Reagent Co., Ltd. (Tianjin, China) and acetone was purchased from Jiangsu Puleisi. Hydrophobic nano-SiO_2_ with an average diameter of 20 nm was obtained from Foshan Lanling Chemical Co., Ltd. (Foshan, China). Polymethyl methacrylate (PMMA) was procured from Taobao. All chemical reagents were used as received without further treatment.

### 2.2. Laser Treatment

**Figure 1:**
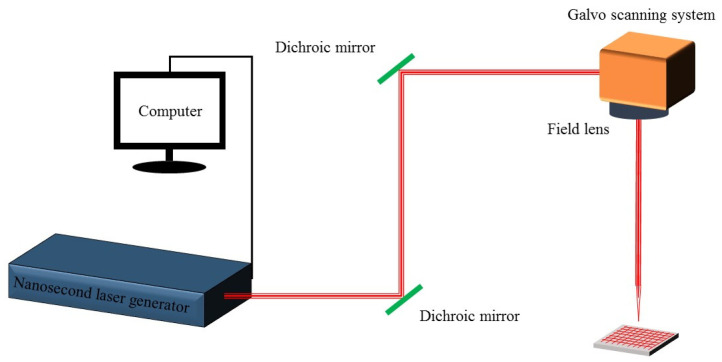
Processing principle and scanning path.

The principle and scanning path of nanosecond laser processing are shown in Figure 1. The processing principle of the laser is that the laser beam emitted by the laser is reflected in the optical path, reaches the galvo scanning system, and then focuses on the sample with the field lens. The field lens adjusts the focal length and controls the size of the laser beam, while the galvo scanning system controls the scanning and positioning of the laser beam. The laser used in this experiment has a center wavelength of 1064 nm, a maximum power of 60 W, a repetition rate of 30 kHz, and a pulse duration of 10 ns for the laser (MFP-5W-60W, Maxphotonics, Shenzhen, China). The laser has a focal length of 319 mm, and the diameter of the focus spot is approximately 50 µm. Firstly, the sample was placed horizontally on the sample stage with laser power set to 12 W, and a horizontal x–y grid scan was performed on the aluminum surface at a speed of 20 mm/s with a 100 µm spacing between adjacent points.

### 2.3. Preparation of Solutions for Coating the Aluminum Sheet

Firstly, 1 g of PMMA was magnetically stirred in a 100 mL acetone solution at room temperature for 24 h to obtain the binder. Secondly, a 10 wt% ethanol suspension of hydrophobic nano-sized SiO_2_ particles was prepared. A total of 5 g of the hydrophobic nano-sized SiO_2_ particles were added to 45 g of anhydrous ethanol and ultrasonically dispersed to uniformly disperse the nanoparticles in the suspension.

### 2.4. Impregnation Process

The polished samples were immersed twice in the prepared binder after laser treatment, each time for 5 s with an interval of 5 s. Then, the samples were immersed five times in the prepared suspension of hydrophobic nano-sized SiO_2_ particles, each time for 5 s with an interval of 10 s. Finally, the prepared samples were left to sit for 24 h.

### 2.5. Surface Topography Characterization Methods

Surface morphology of samples and coated samples after laser ablation was characterized using field emission scanning electron microscopy (Sigma300, ZEISS, Carl Zeiss AG, Jena, Germany) at 200×, 500×, and 1000× magnifications and microscopy (HG-1501T, MHAGO, Wuxi, China) at 30× and 130×.

### 2.6. Wettability Characterization

In this study, the wetting behavior of the samples was measured using a WCA measuring instrument (JCJ-360A) to measure the WCA and SA on the surface of the samples. A 10 μL microsyringe was used to drop approximately 5 μL of the test droplet on different locations of the sample surface. The WCA and SA were measured by dropping 3 drops of deionized water and taking the average value as the final measurement result.

### 2.7. Corrosion Test

In this experiment, the corrosion tests of the samples were performed using a 10% mass fraction CuCl_2_ solution for comparative testing. After preparation, the samples were immersed in the CuCl_2_ solution for 3 min, and the corrosion on the sample surface was recorded using a camera. Finally, the samples were removed, and their surface morphology was recorded by washing with deionized water.

### 2.8. Friction Resistance Test

In this research, the mechanical robustness of the superhydrophobic aluminum surface samples was tested using a sandpaper-based shear wear test (ASTM D4060). The shear wear test involved dragging the superhydrophobic aluminum sample at a constant speed of 1 cm·s^−1^ in one direction on a 2000-grit SiC paper under pressures of 5000 Pa and 10,000 Pa. The water WCA was measured after every 10 cm of wear distance as a function of wear cycles, and the data were plotted accordingly.

## 3. Results and Discussion

### 3.1. Surface Morphology of Aluminum after Laser Treatment

**Figure 2:**
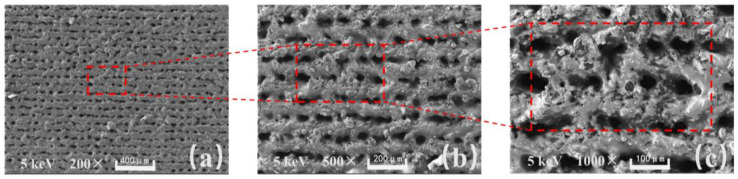
SEM of aluminum surface after nanosecond laser treatment: (**a**) 200 times. (**b**) 500 times. (**c**) 1000 times.

In Figure 2a, the sample was magnified 200 times, and under the intense nanosecond laser ablation, micrometer level grooves were etched onto the surface of the aluminum sheet with micro-pits formed at the intersections. In Figure 2b, the sample was magnified 500 times, and the micrometer level protruding structures on the surface can be more clearly observed. The micrometer level protruding structures are stacked by particles because, in the process of laser action, the momentarily increased energy melts the aluminum and splatters it onto nearby areas. In Figure 2c, the sample was magnified 1000 times, and the micrometer level protruding structures can be observed more clearly. The traces of pulse laser action can be seen inside the micro-pit area, and the density of splashing and melting particles near the micrometer level protruding structures increases at the sub-micron level. The surface of the micrometer-level protruding structures also forms an obvious hairy morphology.

**Figure 3:**
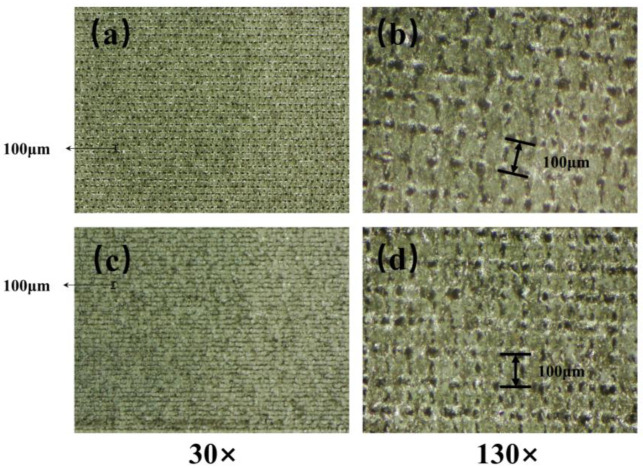
Surface appearance under microscope. (**a**) The aluminum surface after laser ablation at 30 times magnification. (**b**) The aluminum surface after laser ablation at 130 times magnification. (**c**) The superhydrophobic aluminum surface at 30 times magnification. (**d**) The superhydrophobic aluminum surface at 130 times magnification.

Based on the results of the microscopic morphology tests (from Figure 2 and Figure 3), it can be observed that the distance between the two adjacent micro-scale protruding structures on the aluminum surface processed with a nanosecond laser at a scanning speed of 20 mm/s is about 100 µm, and their height reaches approximately 50 µm. The distance between them is relatively large, making it easy for nano-sized SiO_2_ particles to enter and penetrate under the influence of ultrasonic dispersion. Furthermore, a large quantity of nano-sized SiO_2_ particles adhere between those structures. The air in the formed gaps can maintain the maximum surface tension of water droplets, effectively preventing water penetration. Therefore, it can exhibit excellent superhydrophobicity with a high WCA and a low SA.

### 3.2. Wettability Results

**Figure 4:**
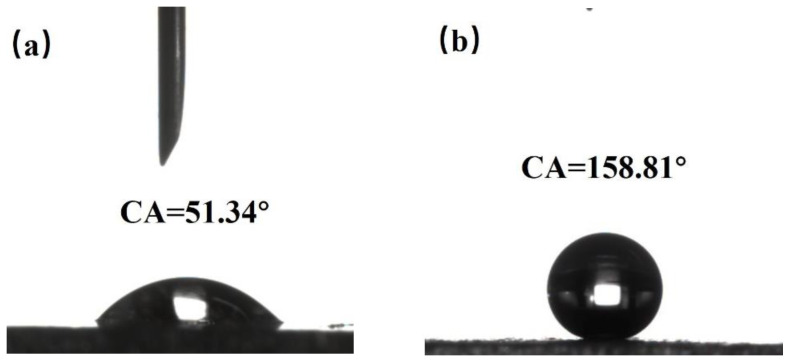
(**a**) The WCA of the original aluminum surface. (**b**) The superhydrophobic aluminum surface.

By using a WCA goniometer, it can be observed that the WCA of the original aluminum surface is 51.34° with hydrophilicity demonstrated in Figure 4a. When measuring the surface of the sample treated with nanosecond laser irradiation showing grid-like patterns with a spacing of 100 μm, it is found that the droplet quickly spreads out on the surface upon contact, exhibiting a WCA of only 0°, indicating superhydrophilicity. This phenomenon can be explained using the Wenzel model [29] where the WCA on object surfaces is related to the surface roughness and surface energy. When the object surface is hydrophilic, the larger its roughness, the smaller its WCA. Conversely, when the object surface is hydrophobic, the larger its roughness, the larger its WCA. For a superhydrophobic aluminum surface, it is difficult for droplets to adhere to the surface. When a droplet is dropped onto the surface of the sample with a micro-injector, the needle-like vibration caused by the drop often leads to a quick rolling of the droplet on the surface. Its WCA is measured at 158.81°, as shown in Figure 4b, and its SA is less than 5°. The droplet is easily released from the surface, indicating excellent superhydrophobicity.

### 3.3. Self-Cleaning Performance

**Figure 5:**
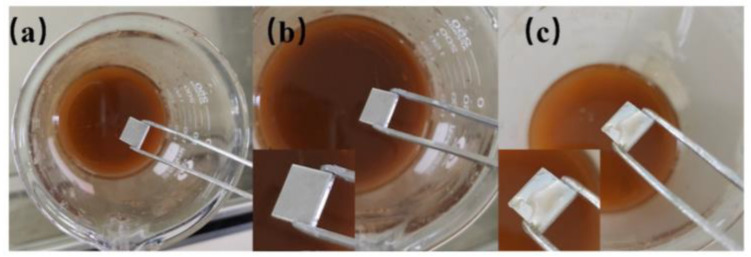
(**a**) Image of superhydrophobic aluminum surface before immersion. (**b**) Image of superhydrophobic aluminum surface after immersion. (**c**) Image of bare aluminum surface after immersion.

**Figure 6:**
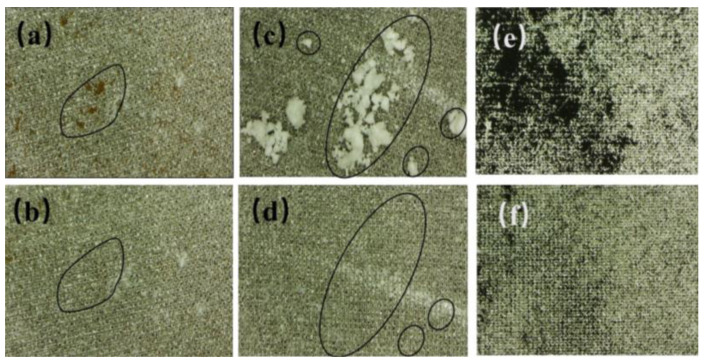
Comparison of self-cleaning ability of substances that can be wetted and substances that cannot be wetted. (**a**,**b**) Dust. (**c**,**d**) Mixture of calcium carbonate and calcium sulfate. (**e**,**f**) Graphite powder.

Figure 5 demonstrates the self-cleaning ability of the superhydrophobic aluminum surface immersed in turbid water. As shown in Figure 5, the bare substrate is contaminated, whereas the superhydrophobic aluminum surface remains clean after being removed from the muddy water. Figure 6 displays photographs of the superhydrophobic aluminum surface contaminated with infiltrating substances (dust, calcium carbonate, and calcium sulfate mixture) and non-infiltrating substances (graphite powder). It should be noted that natural graphite is a hydrophilic material whose wettability is mainly influenced by the presence of hydrocarbons in the air. Graphite powder stored outside will shift from hydrophilicity to hydrophobicity [30,31,32]. By comparing the photos before and after self-cleaning, the rolling trajectory of droplets can be observed.

During the process of the contact and rolling of water droplets on the superhydrophobic aluminum surface, it can be observed that pollutants are mainly removed from the superhydrophobic aluminum surface via two pathways: transferring to the interior of the droplets through particle transference or adhering to the droplet surface through particle adsorption. The pathway through which particles are removed from the superhydrophobic aluminum surface depends on whether the particles are wetted or not. Fully wetted particles are transferred to the interior of the droplets, while non-wetted particles adhere to the droplet surface. Furthermore, as shown in Figure 6, some tiny particles remained on the droplet surface after it carried away the surface dust. This is because these particles have a diameter larger than the pore diameter of the superhydrophobic aluminum surface such that the droplet is too large to contact the tiny particles. By reducing the droplet volume or by dropping the droplet at a certain height multiple times, the small particle pollutants can be removed from the surface. Finally, through observation, it is found that when the superhydrophobic aluminum surface is placed at a certain inclination angle, it does not require a larger kinetic energy for the droplet to carry away the particle pollutants. However, when the superhydrophobic aluminum surface is placed horizontally, the droplet needs to be given an initial kinetic energy (by dropping the droplet at a certain height). When the droplet has a low initial kinetic energy, rolling is observed to be effective at removing pollutants, while for droplets with a higher energy, bouncing is observed to be more effective at removing pollutants. Under the same operational conditions, it is found that pollutants that can be wetted (dust, a mixture of calcium carbonate, and calcium sulfate) have a higher self-cleaning efficiency than those that cannot be wetted (graphite powder). The size of the particles and their wetting properties have a direct impact on the result.

Through these experiments, we have found that the prepared superhydrophobic aluminum surface can effectively remove different types of particle pollutants, demonstrating its excellent self-cleaning performance. We have discovered that the main factors affecting the self-cleaning efficiency of the superhydrophobic aluminum surface include the water WCA, solid surface free energy, the chemical nature of pollutants, the impact pressure and velocity of the droplets, the tilt angle, particle–particle interactions, the thickness of the pollutant layer, particle adhesion to surfaces, particle porosity, etc. [33].

### 3.4. Repeatability and Durability of Superhydrophobic Aluminum Surface

To verify the reproducibility and durability of the superhydrophobic aluminum surface fabricated by utilizing nanosecond laser ablation combined with hydrophobic SiO_2_ nanoparticles, numerous experiments were conducted in this study, particularly to investigate their applicability for large-scale manufacturing.

**Figure 7:**
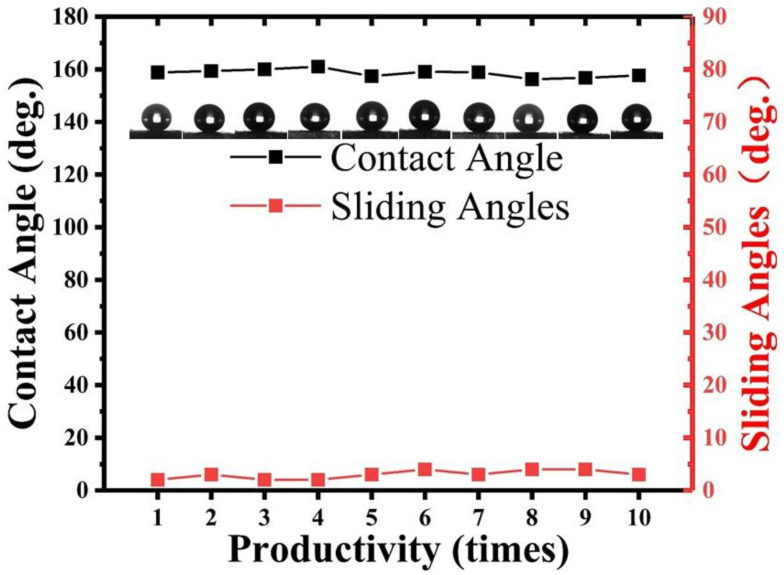
WCA and SA of superhydrophobic aluminum surface under repeated preparation processes.

Reproducible experiments were performed using the same laser parameters, coating parameters, and preparation conditions, wherein one sample was prepared each time, and the static WCA and SA were measured three times at random positions to obtain the mean value. As shown in Figure 7, all the samples prepared in the 10 trials exhibited a static WCA greater than 155° and an SA less than 5°, confirming the reliability and repeatability of this research’s fabrication technique.

Furthermore, after six months, the experimental samples with the same parameters as the initial ones still maintained their superhydrophobicity, and the liquid droplets remained difficult to stay on the micro-tilted surface, indicating the durability of this method.

To verify the method’s applicability for large-area fabrication, 10 mm × 10 mm and 20 mm × 20 mm superhydrophobic samples were prepared, and the same fabrication process as that for the 5 mm × 5 mm samples was demonstrated. The results showed that the WCA and SA exhibited for all the prepared surface areas resembled those of the 5 mm × 5 mm samples, demonstrating the large-scale manufacturability of the superhydrophobic aluminum surfaces fabricated in this research. The results fully verify the reliability and effectiveness of this research method, which provides strong guarantees for practical applications.

### 3.5. Anti-Corrosion Performance of Superhydrophobic Aluminum Surface

**Figure 8:**
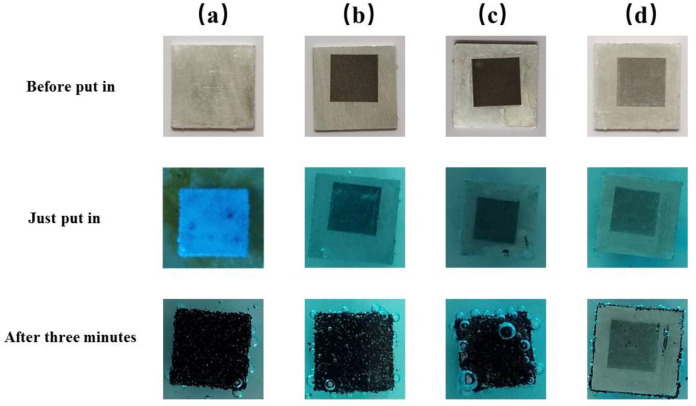
Corrosion process comparison diagram. (**a**) The PAS. (**b**) The LPAS. (**c**) The LPAS and PMMA. (**d**) The superhydrophobic aluminum surface.

To compare the corrosion resistance of the superhydrophobic aluminum surface, the polished aluminum sheet (PAS), the laser-processed aluminum sheet (LPAS), and the aluminum plate treated with PMMA after laser processing (LPAS and PMMA) were immersed in a 10% CuCl_2_ solution together with the superhydrophobic aluminum surface. Figure 8 shows the corrosion process of the PAS, the laser-processed aluminum plate, the aluminum plate treated with PMMA after laser processing, and the superhydrophobic aluminum surface after immersion in a 10% CuCl_2_ solution.

**Figure 9:**
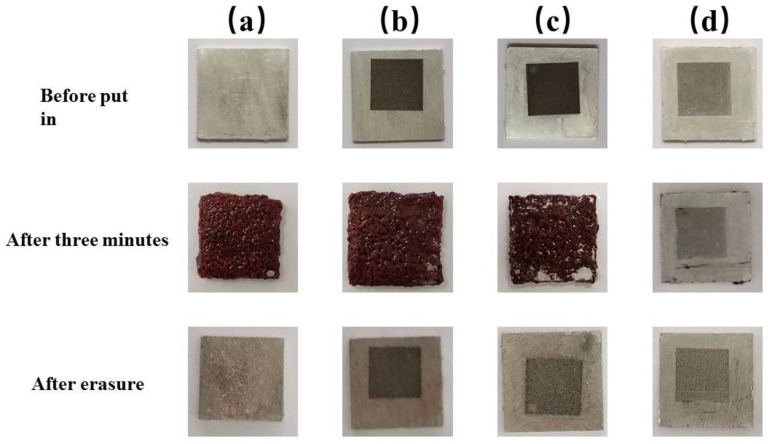
Comparison chart of corrosion results. (**a**) The PAS. (**b**) The LPAS. (**c**) The LPAS and PMMA. (**d**) The superhydrophobic aluminum surface.

As shown in Figure 8, when immersed in a 10% CuCl_2_ solution, the PAS, the LPAS, and the LPAS and PMMA produces tiny bubbles, and more and more bubbles appear on the surface with red copper gradually precipitating on the surface. However, the superhydrophobic aluminum surface (5 mm × 5 mm) does not produce a red area; that is, no red copper precipitates, indicating that the superhydrophobic aluminum surface has anti-corrosion properties. The principle is that the presence of a hydrophobic layer effectively separates the CuCl2 solution from direct contact with the aluminum plate. This phenomenon can be clearly observed when the superhydrophobic aluminum surface first enters the solution. After immersing all the experimental samples in a 10% CuCl_2_ solution for 3 min, they were ultrasonically cleaned one by one. The cleaned samples are shown in Figure 9, from which it can be seen that there is no trace of corrosion on the superhydrophobic aluminum surface, which can be concluded by comparing with other samples. In addition, comparing the wettability of the superhydrophobic aluminum surface before and after corrosion, it can be found that the WCA has not changed, and the SA has slightly increased by about 6°.

(1) $$3Cu^{2+}+2Al\to 2Al^{3+}+3Cu$$

(2) $$Cu^{2+}+2H_{2}O\to 2H^{+}+\mathrm{Cu}{\left(\mathrm{OH}\right)}_{2}$$

(3) $$6H^{+}+2Al\to 2Al^{3+}+3H_{2}$$

The corrosion mechanism can be explained with Equations (1)–(3). The initial step involves the substitution of surface aluminum with Cu^2+^ ions, resulting in the formation of copper (Cu), which subsequently gets deposited over the sample surface. Upon completion of the reaction, the corroded surface turns red due to the presence of copper on the top of the sample surface. Then, the H^+^ generated by hydrolyzing Cu^2+^ and Al^3+^ can react with aluminum to produce hydrogen gas. During this reaction, bubbles can form on the aluminum surface. All of these prove that the superhydrophobic aluminum surface prepared in this study has corrosion resistance in a 10% CuCl_2_ solution.

### 3.6. Mechanical Properties of Superhydrophobic Aluminum Surfaces

**Figure 10:**
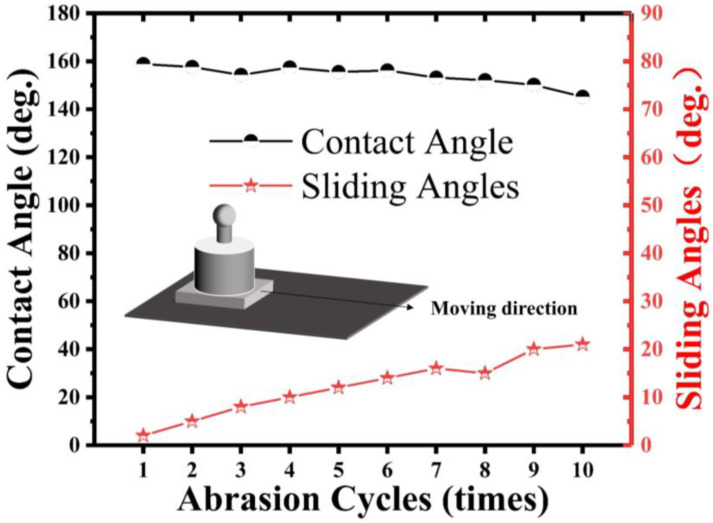
Function diagram and wear test diagram of WCA and SA of superhydrophobic aluminum surface with wear length.

Following the ASTM D4060 procedure, a sandpaper-based shear abrasion test was used to test the mechanical performance of the superhydrophobic aluminum samples and the superhydrophobic samples without micrometer-scale structures (not laser processed). As shown in Figure 10, the shear abrasion test involved dragging the superhydrophobic aluminum sample along one direction on a 2000-grit SiC sandpaper at a constant speed of l cm/s under a pressure of 5000 Pa. The water WCA was measured after every 10 cm of wear distance and plotted as a function of the wear distance.

In Figure 10, we observe the variation of the WCA and SA of a superhydrophobic aluminum surface under different wear distances. Due to the changes in the surface roughness and constant pressure load, the mechanical wear gradually reduces the WCA and increases the SA. For our experiment, we utilized superhydrophobic aluminum samples with micro-nanostructures, which were subjected to 5000 Pa pressure and moved horizontally on 2000-grit SiC paper uniformly. As the wear distance increased, we noticed a gradual reduction in the WCA and an accompanying increase in the SA. However, the sample was able to maintain its superhydrophobicity until 100 cm even under a pressure of 5000 Pa. However, when the load was increased to 10,000 Pa, the superhydrophobic property showed a sharp decline.

**Figure 11:**
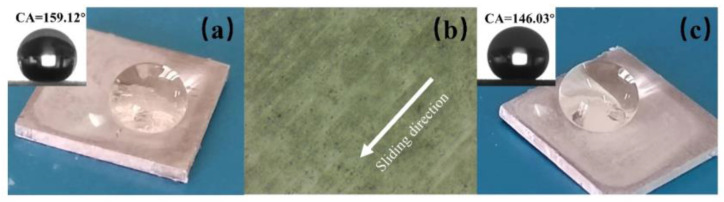
(**a**) The WCA of superhydrophobic samples without micro-structures before wear. (**b**) The superhydrophobic sample surface without micro-structures under a microscope after wear. (**c**) The WCA of superhydrophobic samples without micro-structures after wear.

Furthermore, we also conducted separate tests on the superhydrophobic samples with and without micro-structures (i.e., those that had not undergone laser processing). The results showed that the superhydrophobic properties of both samples were similar; however, in shear-wear tests based on sandpaper, the sample without micro-structures exhibited poor mechanical performance. In this experiment, we placed the non-laser processed superhydrophobic sample under 5000 Pa pressure and moved it horizontally on 2000-grit SiC paper uniformly. As shown in Figure 11, as the wear progressed, the surface coating was scratched, resulting in a rapid decrease in the WCA and a significant increase in the SA. After a single wear, the sample lost its superhydrophobicity. These findings confirm that the presence of micro-structures in our study improves the mechanical performance of the superhydrophobic aluminum surface.

Under a constant pressure load (P), the relationship between the friction force (F) between the sample and sandpaper can be expressed as F = μP. The friction coefficient μ is a function of sliding velocity, normal pressure, and temperature [34]. Keeping the sliding velocity, sandpaper grit, and temperature constant, the magnitude of the friction force is only related to the pressure load in this shear wear test. Therefore, by excluding the influence of sliding speed, sandpaper grit, and temperature on μ, the magnitude of the friction force is only related to a high-pressure load.

## 4. Conclusions

The present study employed nanosecond laser ablation technology to fabricate micrometer-scale surface structures on an aluminum sheet followed by the preparation of a superhydrophobic surface through the combination of PMMA and hydrophobic nano-SiO_2_ particles applied onto this layer. The maximum WCA achieved was 158.81°, while the surface ensured an SA of less than 5°. The experimental results demonstrated the exceptional self-cleaning ability of the prepared superhydrophobic aluminum surface, enabling it to effectively remove two types of wetting substances. During the self-cleaning process, pollutants with varying initial energies were observed to be eliminated through distinct mechanisms. Additionally, the superhydrophobic aluminum surface exhibited remarkable resistance against corrosion, confirming its durability and stability. Notably, this study revealed that the micro–nano double-layered structure endowed the superhydrophobic surface with superior mechanical wear resistance compared to a counterpart lacking micro-scale structures; even after undergoing multiple cycles of 5000 Pa abrasion, it remained stable for an extended period, exceeding ten times that of non-microstructured superhydrophobic surfaces. These findings highlight the robust anti-wear properties inherent in superhydrophobic aluminum surfaces and their potential for enhancing mechanical performance, which may have wide-ranging applications.

## Figures and Tables

**Table 1:** 1060 Aluminum content (mass fraction, %).

Al	Fe	Cu	Mn	Mg	Si	Ti	V	Zn
99.6	0.35	0.05	0.03	0.03	0.25	0.03	0.05	0.05

## Data Availability

The datasets generated during and/or analyzed during the current study are available from the corresponding author upon reasonable request.
